# Genome-Wide Characterization of DNA Demethylase Genes and Their Association with Salt Response in *Pyrus*

**DOI:** 10.3390/genes9080398

**Published:** 2018-08-06

**Authors:** Chunxiao Liu, Hui Li, Jing Lin, Ying Wang, Xiaoyang Xu, Zong-Ming (Max) Cheng, Yonghong Chang

**Affiliations:** 1 Institute of Pomology, Jiangsu Academy of Agricultural Sciences/Jiangsu Key Laboratory for Horticultural Crop Genetic Improvement, Nanjing 210014, China; chuntianxiaozi@163.com (C.L.); lihui7904@163.com (H.L.); lj84390224@126.com (J.L.); 2016104047@njau.edu.cn (Y.W.); 2 Department of Plant Sciences, University of Tennessee, Knoxville, TN 37996, USA; 3 Institute of Botany, Jiangsu Province and Chinese Academy of Sciences, Nanjing 210014, China; intergoogle@126.com

**Keywords:** *Pyrus betulaefolia*, demethylase genes, genome-wide, salt response

## Abstract

DNA methylation plays important roles in genome protection and the regulation of gene expression and it is associated with plants’ responses to environments. DNA demethylases are very important proteins in DNA methylation regulation. In this study, we performed genome-wide and deep analysis of putative demethylases (DMEs) in pear. Seven *DME* genes were found in the pear genome and were defined as *PbDME1–7* based on their domain organization. Results were supported by the gene structural characteristics and phylogenetic analysis. The gene structure of the *DME* genes were relatively complex and the DME7 proteins didn’t contain the Perm_CXXC domain. The *DME* genes experienced a whole genome duplication event (WGD) that occurred in the ancestor genome of pear and apple before their divergence based on the *K*s values. Expression results showed that high salinity stress could influence the expression level of *DME*s and salt-responsive genes in *Pyrus betulaefolia*. Furthermore, the methylation levels of salt-responsive genes changed under salt stress treatment. Results suggested important roles of *PbDME* genes in response to salt stress and are useful for better understanding the complex functions of this *DME* genes, which will facilitate epigenetic studies in pear trees salt tolerance.

## 1. Introduction

DNA methylation is an important and conserved epigenetic mark that is present in animals and plants, and it is important for genome integrity, development, and environmental responses [1,2]. Since it was first coined in 1942 by Conrad Waddington [3], epigenetics has become a hot research topic. DNA methylation predominantly occurs at cytosines in a symmetric CG sequence context in animals; oppositely, methylation in plants commonly occurs in an asymmetrical CHH sequence context (H = A, C, or T), as well as in the symmetrical CG and CHG contexts [4,5]. These modifications are mainly performed by 3 cytosine-5 DNA methyltransferase (C5-MTase) families, including methyltransferases (METs), chromomethyltransferase (CMT), and domains-rearranged methyltransferases (DRMs). The final level of DNA cytosine methylation is dynamically regulated mainly by the common action of both DNA methyltransferases and demethylases [6].

DNA methylation can be actively removed by DNA demethylases [7]. Active DNA demethylation in plants is initiated by a family of 5-meC DNA glycosylases/lyases (i.e., DNA demethylases (DME)) [8,9,10]. In *Arabidopsis*, four proteins of the DME family with demethylase activity have been identified. They are: repressor of silencing 1 (ROS1), demeter (DME), demeter-like 2 (DML2), and demeter-like 3 (DML3) [4]. Genetic and biochemical studies have revealed that the *Arabidopsis* DNA glycosylase domain-containing proteins ROS1 and DME initiate erasure of 5-methylcytosine through a base excision repair process. The *Arabidopsis* genome encodes two paralogs of ROS1 and DME, referred to as DML2 and DML3 [11]. ROS1 is the first genetically characterized DNA demethylase (the first enzyme in the active DNA demethylation pathway) in eukaryotes [12]. ROS1 can remove the 5-meC base and nick the DNA backbone, leaving a single nucleotide gap that is filled with an unmethylated cytosine through a base excision repair pathway [13,14]. DME is required to demethylate regions of DNA, as part of the base-excision repair (BER) pathway [15] is required for the maternal allele-specific expression of imprinted genes in the central cell and endosperm [16]. Silencing of these genes caused an increased level of DNA cytosine methylation in all sequence contexts at specific genomic loci [9]. In general, active demethylation, which involves enzymatic action of DNA demethylases that can excise 5-meC from all sequence contexts, is achieved by a base excision repair pathway [7,9]. Additionally, studies have performed genome-wide analysis of the characteristics of the DNA demethylases in different species, such as *Solanum lycopersicum* [6] and peanut [17]. Genome-wide analysis is a common and popular method to elucidate family genes in a comprehensive way. For example, it revealed the presence of essential roles of *Arabidopsis COP9* genes in the signalosome [18], and genome-wide identification and analysis of rice genes showed conserved relationship and between *Oryza sativa* L. subsp. *japonica* and *Oryza sativa* L. subsp. *indica* cultivars, and low-temperature stress at the vegetative growth stage [19], and comprehensive identification and analysis of *PYL* genes in *Brassica napus* explained the abiotic stress response [20].

Besides the important role that DNA demethylases play in plant development, growing evidence has suggested that plant responses to environment stresses is also closely related to the level of DNA demethylation [21,22,23,24]. In recent years, besides model plants *Arabidopsis thaliana* [25] and rice [26,27,28], many other plant species have been used to study DNA methylation under abiotic stress, such as cotton [29], sorghum [30], soybean [31], tomato [32], and *Populus. trichocarpa* [33], which suggested that DNA methylation plays an important role in regulating plant adaptation to environmental stress. For instance, exposure to biotic stress such as pathogen attack leads to dynamic methylation changes across the *Arabidopsis* genome [34]. DNA demethylases could target promoter transposable elements to positively regulate stress-responsive genes in *Arabidopsis* [35]. In *A. thaliana*, mutants lacking the *SOS1* transporter, which changed DNA methylation level under salt stress, showed extreme sensitivity to salt stress, and had various defects of Na^+^ efflux [36,37]. Aluminum [38], heavy metals [39], and water stress [40] can cause an increase or decrease in cytosine methylation throughout the genome, and at specific loci. In rape, cadmium stress stimulated demethylation at specific loci, according to the methylation-sensitive amplification polymorphism (MSAP) approach [41]. An overexpressed *NtROS2a* gene with significant similarities to *Arabidopsis ROS1* could increase the tolerance of tobacco to various abiotic stresses [42]. Treatment with 5-azacytidine (5-azaC), a demethylating agent, could replace low-temperature treatment in several vernalization-requiring plant species [43,44]. There were also other salt-related genes reported in different species, which played important roles in the salt response, such as *OsSta2*, which enhanced salt stress tolerance in rice [45], salt-related genes could affect fruit antioxidant systems in tomato [46], and salt stress could also affect ion concentration, proline content, antioxidant enzyme activities in tomato [47]. Genetic and epigenetic factors played crucial roles in the control of plant salinity, drought, cold, heat, and other stress responses [24]. These facts implied the involvement of epigenetic mechanisms in the regulation of environmental stresses.

Pears (*Pyrus* spp. L.) are one of the most important fruit crops in the family of *Rosaceae*. It is the third most important fruit crop in temperate zones, after grape and apple [48,49]. The *Pyrus bretschneideri* genome was released, which provides a rich resource as a reference genome and for comparative genomic analyses [49]. Salt stress is one of the major abiotic stresses in agriculture worldwide that causes crop failure by interfering with the profile of gene expression and cell metabolism. The production of pears is quite limited due to the spread of soil salinization. Considerable effort has been directed at investigating salt stress in pear plants, especially its accumulation, transduction, and physiological and metabolic effects [14,50,51]. *Pyrus betulaefolia* Bunge is a Chinese native species and is commonly used as a rootstock in pear orchards. Pear trees show improved salt tolerance, yield, and economic efficiency in saline conditions after grafting onto this rootstock [48,51,52]. The *P. betulaefolia* could reduce the Na^+^ absorption from the soil and limit its transport to shoots (the scion) [51,52]. Although the salt stress signal transduction pathway has been intensively studied, it is especially unclear as to what the role of DNA methylation is. In the present study, we performed a comprehensive analysis of the DNA demethylase gene family in pear, including a phylogenetic tree, chromosomal localization, gene structure, synteny analyses, the expression profiles of these genes under salt stress, as well as investigations into 10 salt-induced genes and detections of the level of methylation for five promoter regions of the salt-induced genes. The results will help with future investigations of the roles of demethylases in improving plant salt tolerance aimed at the functional characterization of stress tolerant-related genes, which can be utilized for the genetic improvement of pear trees, and for studies on their cultivation in high salt areas.

## 2. Materials and Methods

### 2.1. Identification and Chromosomal Mapping

Whole genome annotation sequences of pear (*P. bretschneideri*) were collected from the pear genome project (http://peargenome.njau.edu.cn/) [49], and RNA sequences were collected from the National Center for Biotechnology Information (NCBI) database (http://www.ncbi.nlm.nih.gov/). The *Arabidopsis thaliana*, *Medicago truncatula*, *Vitis vinifera*, *Brassica rapa*, *Glycine max*, *Malus domestica*, *Prunus persica,* and *Solanum lycopersicum* gene files were downloaded from Phytozome v11.0 (http://www.phytozome.net/). The *Arabidopsis* expression data of different organs [53] and salt stress [54] were downloaded on line. The Hidden Markov Model (HMM) profile of the RNA recognition motif-DME (RRM-DME) domain (PF15628) was obtained from the Pfam website (http://pfam.xfam.org/), and it was employed as a query to identify all possible demethylases using HMMER (V3.0) software (HHMI Janelia Farm Research Campus, 19700 Helix Drive, Ashburn, VA 20147, USA) [55]. To validate the HMM search, all candidate sequences were used as queries to search the NCBI non-redundant (nr) protein database with the blastp program, and the results with the best RRM-DME hits were retained for further analysis. The demethylase sequences were confirmed based on the presence of an RRM-DME domain, and all of the putative demethylases proteins were aligned to tomato and peanut demethylase proteins to classify them into different groups [6,17].

Positional information of all the demethylases was parsed from the pear genome; the locations of them in pear was drafted using MapInspect software (version 1.0) (http://mapinspect.software.informer.com/).

### 2.2. Protein Properties and Sequence Analyses

Protein properties, including the molecular weights (MWs) and isoelectric point (pIs) of the DMEs, were predicted using the online tool Compute pI/Mw7 [56]. The motif analyses of the DMEs were detected using MEME online software [57] with the default parameter settings, except that the width of motifs was set from 6 to 50, and the minimum number of motifs was 2 and the maximum was 15. The gene structures of the DMEs were parsed from the general feature format (GFF) files of the pear genome database, and diagrams of the exon-intron structures were drawn using the online program Gene Structure Display Server [58] (GSDS; http://gsds.cbi.pku.edu.cn/).

### 2.3. Multiple Sequences Alignment and Phylogenetic Analysis

A multiple alignment of the DME protein sequences from pear, *A. thaliana*, *B. rapa*, *G. max*, *M. domestica*, *M. truncatula*, *V. vinifera*, *P. persica,* and *S. lycopersicum* were constructed with ClustalX 2.0 [59], and gaps and poorly aligned sections were removed. Phylogenetic trees were generated using the neighbor-joining method in MEGA7 [60] software, and the reliability of the interior branches was assessed with 1000 bootstrap re-samplings.

### 2.4. *K*s Calculation and Divergence Time Estimation of Homologous Gene Pairs

The ratio of non-synonymous substitutions (*K*a)/synonymous substitutions (*K*s) was evaluated to determine homologous relationships and divergence time of *DME* genes. *K*a and *K*s values, and the ratio of *K*a/*K*s of *PbDMEs* homologous gene pairs, and orthologous gene pairs between pear and apple were calculated using DnaSP v5 [61]. The approximate divergence time (T) of the *DME* homologous gene pairs in pear, apple, or between them were calculated based on the formula T = *K*s/2λ assuming a clock-like rate (λ) of 9.26 synonymous substitutions per 10^9^ years [49]. A syntenic diagram was constructed using Circos software [62].

### 2.5. Plant Materials and Stress Treatments

*Pyrus betulaefolia* Bunge, a Chinese native species of pear commonly used as a rootstock, was used for salt stress expression analysis. *P. betulaefolia* plants were harvested from the tissue cultures of one-month seedlings and then transferred into soil after roots generated. At the eight-leaf stage after about 45 days, the roots of the *P. betulaefolia* plants were then immersed into solution with 200 mM NaCl and the deionized water as controls. Roots, stems and leaves were collected at 0 hr, just prior to the application of the salt treatment, and then at 12, 24, 48 and 72 h after the salt treatment. Collected samples were immediately frozen in liquid nitrogen and stored at −80 °C. The experiments were repeated three times, and each experiment was comprised of 12 plants per treatment. The presented data represents the mean ± the standard error of three biological replicates.

### 2.6. RNA Isolation and Reverse Transcription-Quantitative PCR

Total RNA was isolated using a plant RNA purification kit (MoLFarming, Cat. No. RK16-50 T, Nanjing, China) from leaf tissues according to the manufacturer’s instructions. A *t*-test was used for statistical analysis. The expression of DMEs and salt-induced genes was analyzed using an ABI 7500 real-time PCR system (Applied Biosystems, Carlsbad, CA, USA) with the SYBR Green Master Mix (TaKaRa, Dalian, Liaoning, China). Gene-specific primers were designed based on the gene sequences using Primer Premier 5.0 (Carnegie Institute of Washington, Washington, US). *P. betulaefolia* EF1α (*Pbr034452.1*) was used as internal controls for normalization. The amplification parameters were as follows: 95 °C hold for 10 min, followed by 40 cycles at 95 °C for 15 s, 60 °C for 15 s, and 72 °C for 15 s. For the melting curve stage, the default settings were chosen. Nonspecific products were identified by inspecting melting curves. Experiments for three technical replicates for each biological replicate were carried out. All of the primers used in this paper have been listed in an additional table: Table S1. The RNA integrity/quality and quantitative PCR (qPCR) primer efficiency were illustrated in Figure S1.

### 2.7. Bisulfite Conversion of Genomic DNA and Methylation Sequencing

Genomic DNA was extracted a using Genomic DNA Extraction Kit Ver.5.0 (TaKaRa Code. 9765). Bisulphite sequencing aliquots of 800 ng DNA were treated with sodium bisulphite using the EpiTect Fast Bisulfite Kit (QIAGEN, Cat. 59824, city, state if US, country,) according to the manufacturer’s instructions. DNA was amplified by PCR with EpiTaq™ HS (Takara Code No. R110). PCR products were cloned into the pMD19-T Simple Vector (Takara Code No. 3271) and the clones were sequenced. The amplification parameters were as follows: 95 °C for 5 min, 40 cycles of (94 °C for 30 s, 542 °C for 30 s, 72 °C for 40 s), and 72 °C for 5 min. For each region, more than 20 independent top-strand clones for were sequenced from each sample.

## 3. Results

### 3.1. Genome-Wide Identification of DMEs in Pear

The whole-genome sequence of pear [49] was used for the genome-wide identification of the *DME* gene family in pear. Using (HMMER v3.0) method [55] with data from a query on the DME family (PF15628), protein databases were searched. A total of seven DMEs were identified in the pear genome, all of which were confirmed by the presence of the RRM-DME domain (Table 1). Subsequently, we did gene ontology (GO) analysis of the seven *PbDME* genes to annotate for their functions (Table S2). Their corresponding protein sequences, coding sequences, and genomic sequences were obtained from the pear genome for further analysis. Subsequently, the structure of the seven putative pear *DME* genes were characterized, and multiple sequence alignments analyzed (Figure S2). Each DME protein contained an RRM-DME domain, which is a fundamental characteristic of DME proteins. The length of the coding sequence of the pear *DME* genes ranged from 3702 to 6048 bp, and their derived proteins varied from 1233 to 2015 amino acids in length. Their MW varied from 135.88 to 225.75 kDa with pI values from 5.89 to 9.01 (Table 1).

### 3.2. Classification and Phylogeny Analysis of the *PbDMEs*

**Figure 1:**
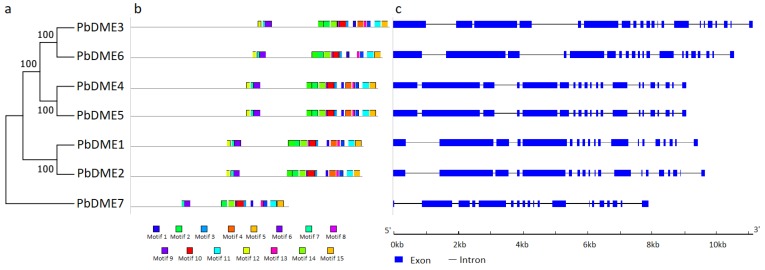
Phylogenetic and structural analysis of the *PbDME* family in pear (*Pyrus bretschneideri*). Phylogenetic tree of the *PbDME* family in pear was generated using the neighbor-joining with 1000 bootstraps in MEGA 7 [60]. Motif analysis was with MEME software [57], and gene structure was analyzed using GSDS 2.0 [58].

**Figure 2:**
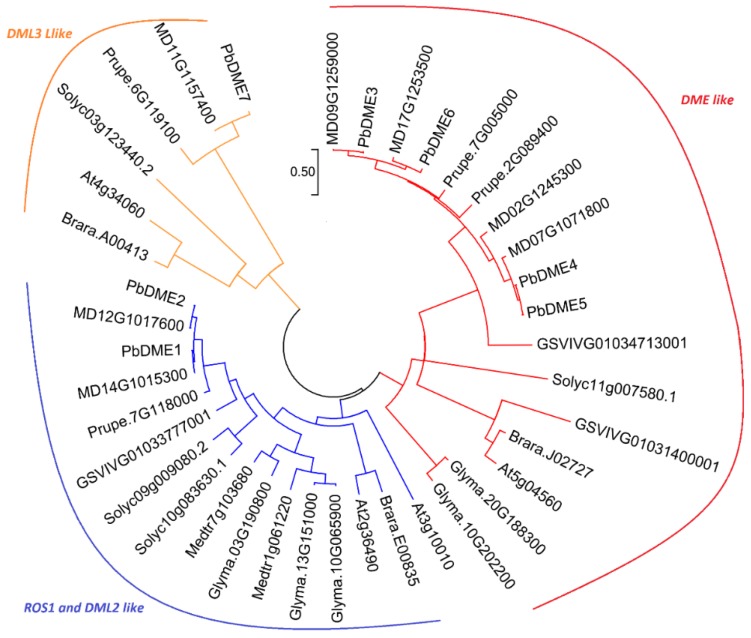
Evolutionary relationships of the *DME* gene family in nine plant species. The evolutionary analysis included the DME genes from *Pyrus. bretschneideri* and eight other species: *Arabidopsis. thaliana*, *Malus. domestica*, *Prunus. persica*, *Brassica. rapa*, *Glycine. max*, *Medicago. truncatula*, *Vitis. vinifera*, and *Solanum. lycopersicum*. Different groups are illustrated in different colors. The evolutionary history was inferred using the neighbor-joining method with 1000 bootstraps in MEGA 7.0 [60].

Based on sequence similarities and the composition of the conserved RRM_DME domain, the seven *DME* genes in pear (*P. bretschneideri*) were identified and designated as *PbDME1–7* (Table 1 and Figure 1a). The RRM_DME domain is a required element for a gene to be a DME, and each of the seven pear *DME* genes contained one RRM_DME domain. There is another short Perm_CXXC domain (PF15629), which is very close to the RRM_DME domain, that was found in six DME members except for *PbDME7* (Figure S2). Other domains may be characteristic of the different types of members within the DME gene family. The seven *DME* genes were mainly classified into three groups, *PbDME1/2* were homologous to *AT2G36490* (*ROS1*) and *AT3G10010* (*DML2*) in *Arabidopsis*; the *PbDME3–6* were homologous to *AT5G045609* (*AtDME*); and the *PbDME7* was homologous to *AT4G34060* (*AtDML3*) (Figure 2). *PbDME* family members were relatively complex in gene size and structure. They all contained more than 18 introns for each member (Figure 1c). Though there was high similarity among different members, they were also divided into three different groups, consistent with that in *Arabidopsis* (Figure 2).

In order to better understand the phylogenetic relationship of the pear *DME* genes, an unrooted phylogenetic tree of *DME* from pear and eight other species was constructed (Figure 2). A phylogenetic tree of 39 *DME*s was generated from the aligned full protein sequence of four demethylases from *A. thaliana*, seven from *M. domestica*, four from *P. persica*, three from *B. rapa*, five from *G. max*, two from *M. truncatula*, three from *V. vinifera*, four from *S. lycopersicum*, and seven from *P. bretschneideri*. Results indicated that all 39 genes were mainly clustered into three groups (Figure 2). This was consistent with the prior established classification based on domain compositions (Figure S2). The different clades of DME may indicate diverse functions that are conserved among species.

### 3.3. Characteristics and Chromosomal Location of Pear *DME* Genes

**Figure 3:**
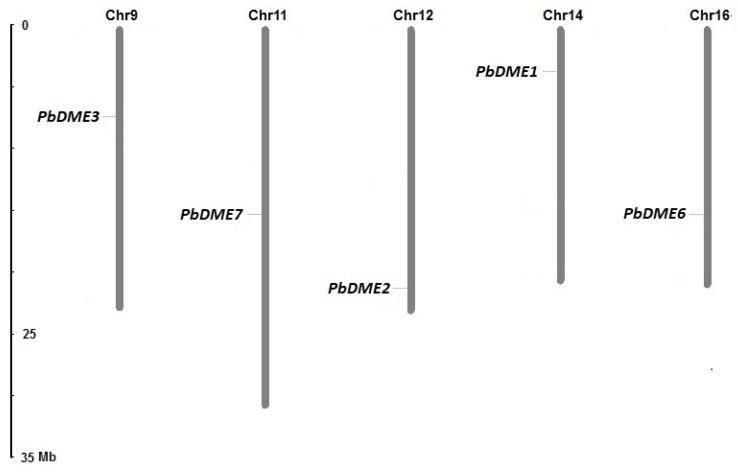
Chromosomal distribution of *PbDME* genes in pear. The Roman numerals on top of each chromosome represent the number of the chromosome. *PbDME4/5* were both located on scaffold404.0. So, they were not included in this map.

The genomic distribution of the identified *DME* genes on pear chromosomes was determined. Five pear *DME* genes were scattered on five of the 17 pear chromosomes, with the exceptions of *PbDME4/5,* which were both located on scaffold404.0. All the five genes were located on five different chromosomes; *PbDME1* on chromosome 14, *PbDME2* on chromosome 12, *PbDME3* on chromosome 9, *PbDME6* on chromosome 16, and *PbDME7* on chromosome 11 (Figure 3). The *DME* genes comprise a small gene family in pear.

The conserved motifs and intron-exon distribution of *PbDME* genes were analyzed to better understand their structural features (Figure 1b,c). The conserved motif analysis of *PbDME*s supported the previously presented phylogenetic relationships and classification of them (Figure 1a,b). Fifteen conserved motifs were detected among the different DMEs. Motifs 1–6 and 14 were detected in all the DME proteins; motifs 7, 9–13, and 15 were absent in the *PbDME7*; *PbDME1* and *PbDME2* contained all the other motifs except for the motifs 12, 13, and 15 (Figure 1b, Figure S3, and Table S3). In general, the number and types of motifs, and the gene structure of the pear *DME*s were consistent with the determined phylogenetic relationship (Figure 1b). 

The intron/exon structure of the pear *DME* genes was analyzed. Results indicated that pear *DME*s have a relatively complex gene structure, with more than 18 introns for each gene (Figure 1c). *PbDME1* and *PbDME2* had 18 introns, while the members of the *PbDME4–7* had 19 introns. *PbDME3* contained the highest number of introns, which was 20 (Figure 1c). Genes originating from the same group, and those that were clustered together in same clade in the phylogenetic tree had similar structural organization (Figure 1).

### 3.4. Evolution of *DME* Family Genes in the Pomoideae

**Figure 4:**
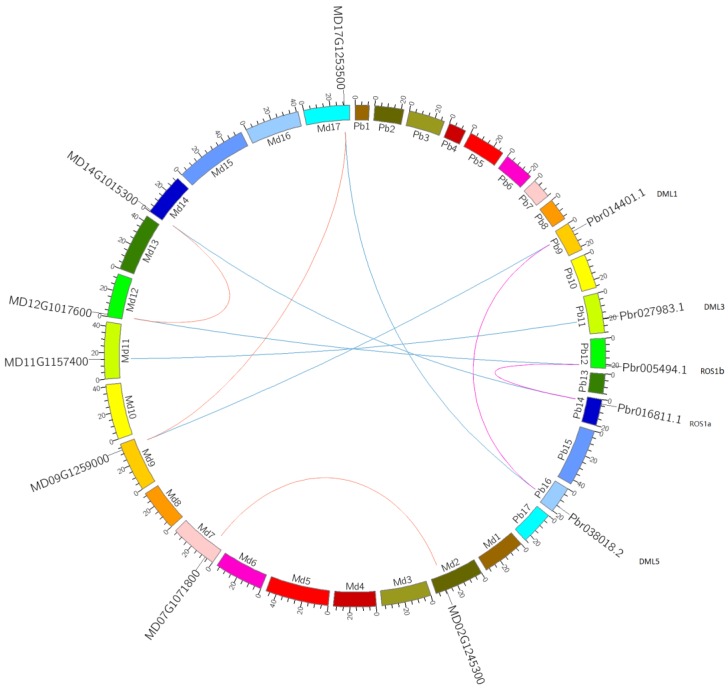
Intra- and interspecific *K*s comparisons of *DME* genes in the pear and apple genome. The pink and orange lines indicate intraspecific synteny of *DME* genes in pear and apple, the blue lines indicated interspecific synteny between pear and apple.

Previous studies have suggested that a recent whole genome duplication (WGD) event shared by pear and apple occurred 30~45 million years ago (MYA). This was prior to the divergence of the two groups 5.4~21.5 MYA, but after their divergence from strawberry [49,63]. An analysis of the relationship between DME homologous gene pairs across pear and apple could provide insights into their divergence and evolution. Therefore, a comparative analysis of the homologous *DME* gene pairs across pear and apple was conducted (Figure 4). Results indicated that there were five orthologous *DME* gene pairs between pear and apple (*PbDME1* & *MD14G1015300*, *PbDME2* & *MD12G1017600*, *PbDME7* & *MD11G1157400*, *PbDME3* & *MD09G1259000*, and *PbDME6* & *MD17G1253500*), two paralogous *DME* gene pairs in pear (*PbDME1* & *PbDME2*, and *PbDME3* & *PbDME6*), and three paralogous gene pairs in apple (*MD09G1259000* & *MD17G1253500*, *MD02G1245300* & *MD07G1071800*, and *MD14G1015300* & *MD12G1017600*) (Figure 4 and Table S4). The divergence time of the orthologous gene pairs between pear and apple was estimated using the *K*s values was in the range of 2.59~5.01 MYA, which was considerably less than that of the speciation time of 5.4~21.5 MYA. It indicates that the divergence of the orthologous gene pairs between pear and apple occurred after their speciation, which was consistent with the conclusions of previous studies [49,63]. Additionally, based on *K*s values, the estimated divergence time of the *DME* paralogous gene within the pear or apple genome was in the range of 8.30 to 121.24, and 8.03 to 11.80, respectively; which both occurred after the WGD event did, in the ancestor of pear and apple [49,63]. This indicates that the *DME* genes in both pear and apple experienced the WGD event. Furthermore, the *K*s values between pear *DME* homologous gene pairs were higher than in apple (Table S4). This may indicate that a more conserved evolutionary rate exists in pear than in apple. Most importantly, no genes may have been lost after the WGD event, indicating the important role that *DME* genes play in pear and apple.

### 3.5. Expression of *PbDMEs* and Salt-Related Genes in Response to Salt Stress

**Figure 5:**
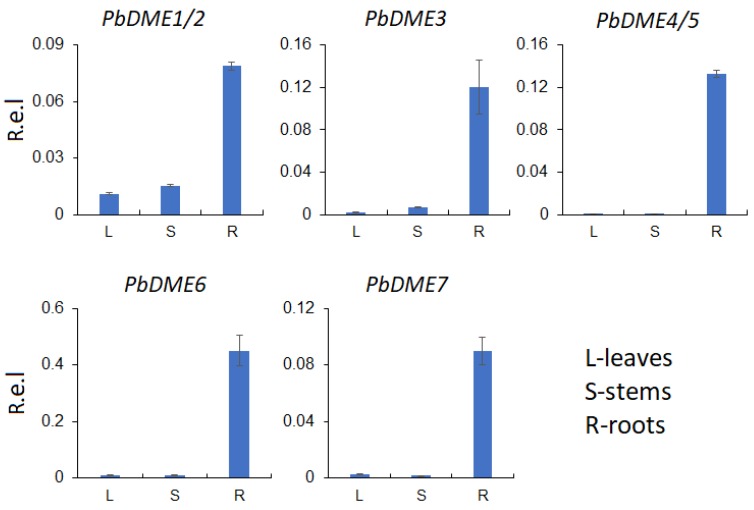
Relative levels of expression of *PbDME* genes in different pear tissues. Level of *DME* gene expression in leaves, stems, and roots of pear as determined by reverse transcription-quantitative PCR (RT-qPCR) using the 2^−∆ ∆ CT^ method. R.e.l indicates relative expression level.

Seven *PbDME* genes were identified in the genome sequence of cultivated pear. To gain insight into the putative function of these genes, their expression patterns in different tissues, including leaves, stems, and roots, were examined. Results showed that the expression of different *DMEs* varied between leaves, stems, and roots (Figure 5). All seven of the *PbDME* genes exhibited their highest expression in roots, where they were expressed seven to 468 times higher than in leaves, and five to 602 times higher than in stems. *PbDME4/5* exhibited the greatest difference of expression level in roots, compared to that in stems and leaves, where the expression level was 468 times higher in roots than in leaves and 602 times higher than in stems. Even the expression levels of *PbDME1/2,* which had the least difference of expression in roots among the seven genes, had 5–7 times higher expression level in roots than in leaves and stems (Figure 5). All of the seven *PbDME* genes exhibited dominant expression in roots. There was a similar situation for *AT2G36490* and *AT3G10010* genes in *Arabidopsis*, but this was different for *AT5G04560* and *AT4G34060* (Figure S4). Results suggest that *PbDME* genes play important roles in roots; which is the key place for the operation of *P. betulaefolia*’s response to environments.

**Figure 6:**
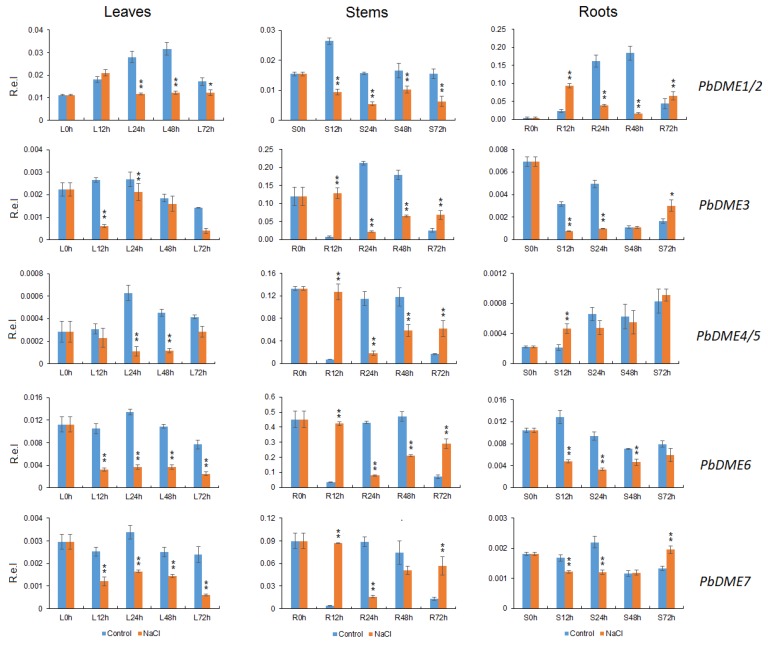
The patterns of expression of *PbDME* genes in different pear tissues in response to salt stress. Young pear plants (approximately eight leaves-old) growing in soil in pots were exposed to 200 mM NaCl by watering the plants with the salt solution. Roots (R) (a), stems (S) (b) and leaves (L) (c) were sampled at 0, 12, 24, 48 and 72 h, and relative expression was determined by RT-qPCR analysis. Relative expression was calculated using the 2^−∆ ∆ CT^ method. R.e.l indicates relative expression level.

Furthermore, the expression of *DME* genes was detected in leaves, stems, and roots of pear trees growing under normal conditions; however, the *PbDME* genes were also induced or repressed in response to salt stress (Figure 6), which was quite different from the *Arabidopsis DME* genes (Figure S5). In leaves, all seven of the *PbDME* genes were down-regulated after salt stress treatment, compared to the controls. In stems, except for *PbDME1/2,* which were down-regulated, *DME*s were significantly induced at 12 h and 72 h under salt stress. In roots, genes *PbDME1/2* and *PbDME4/5* were highly induced at 12 h. However, genes *PbDME3*, *PbDME6,* and *PbDME7* were repressed after salt stress treatment (Figure 6). Results suggested that *PbDME* genes were responsive to salt stress, and that they are indeed involved in salt signal transduction, though the mechanism is still unclear in *P. betulaefolia*. Additionally, the variety of the expressions under salt stress or in different organs for *PbDMEs* indicated that they played important roles in response to salt stress, and that they functioned in various stress regulation pathways.

The expression patterns of several salt-responsive genes were also investigated using reverse transcription-quantitative PCR (RT-qPCR) to gain further insight into their possible function. Ten salt-responsive genes (*PbCBL1*, *PbCIPK1*, *PbCIPK3*, *PbCIPK9*, *PbCIPK13*, *PbCIPK14*, *PbCIPK15*, *PbCIPK18*, *PbCIPK20,* and *PbCIPK22*) were selected to confirm their expression in response to the salt stress treatment that was used to investigate the expression of the pear *DME* genes (Figure S6 and Table S1). Results indicated that the salt-responsive genes were induced or repressed in response to the salt stress utilized in the present study. For example, *PbCIPK9*, *PbCIPK13-16*, *PbCIPK18,* and *PbCIPK22* showed significantly induced expression at 12 h after the salt treatment, while *PbCBL1* showed significantly induced expression at 24 h. *PbCIPK3* was induced gradually over time. On the other hand, the expressions of these genes were repressed after 24 h salt treatment. *PbCIPK20* was expressed less than the control (Figure S6).

### 3.6. Changes in the Level of Methylation of Some Salt-Responsive Genes in Response to Salt Stress

To further investigate the role of *PbDME* genes in regulating gene expression, five salt-responsive genes were selected to determine if the level of methylation in their promoters (which contain CpG islands) changed in response to salt stress. Results indicated that four of the genes (*PbCIPK1*, *PbCIPK20*, *PbCIPK22,* and *PbCBL1*) exhibited demethylation at CpG sites in response to salt stress compared to the level observed in the controls (Table 2). However, there was no difference detected in *PbCIPK3* of the methylation level after salt stress treatment. However, the expression of *PbCIPK22* and *PbCBL1* genes increased at 24 h under salt treatment, and the level of methylation decreased (Table 2 and Figure S6). Previous studies have reported that *PbCBL1* and *PbCIPK2* are involved in the salt overly sensitive (SOS) pathway, which was the first identified calcineurin B-like- calcineurin B-like interacting protein kinases (CBL-CIPK) pathway for maintaining ion homeostasis in plant cells [64,65]. Other complex regulatory pathways involved in salt response may be present in pear. Indeed, the expression of *PbDME* genes were either up- or down-regulated in response to the salt stress treatment (Figure 6), which was consistent with the methylation level of the salt-responsive genes. Therefore, it is suggested that *PbDME* genes play a crucial role in the response to salt stress in *P. betulaefolia*.

## 4. Discussion

### 4.1. Complex Gene Structures May Indicate Various Functions

We identified seven *DME* genes in pear in this study, denominated *PbDME1–7*, which possessed high sequence similarity. Phylogenetic analysis showed that the pear *DME* genes were classified into three groups according the clusters that showed in *Arabidopsis*, which differed in their domain or motif organization (Figure 1 and Figure 2). The domain architecture and intron/exon structure of the *DME* genes in pear were relatively fixed, and they were highly conserved among different members (Figure 1, Figures S2 and S3). All of the PbDME proteins possess one RRM_DME domain, and the other six PbDME proteins also contained one Perm_CXXC domain, except for PbDME7. Additionally, the introns of the seven *PbDME* genes numbered 18 to 20, which was very conserved. The structure and feature of *DME* genes in tomato and *Arabidopsis* [6] were basically the same, demonstrating that they perhaps have similar functions in regulating the level of DNA methylation. Besides the characteristic gene structures and domains, or their functions in abiotic stress response, the *DME* genes had various functions. For example, ROS1-type *DME* genes have a role in UV-B induced- and oxidative DNA damage in *A. thaliana* [66], and they play important roles in its immune responsiveness [67]. *SlDML2*, homologous to the *AtROS1* gene, also plays a role in tomato ripening [10]. The clustering analysis in this study (Figure 2) allows us to infer the DME’s functions in *P. betulaefolia*. On the other hand, the conserved structures of each group, and the differences among DME members suggest the existence of conserved and diverse functions. DMEs may also have undergone specialization during the evolution of this gene family.

### 4.2. *DME* Genes in Other Plants

Plant epigenetics has received considerable attention in both basic and applied research in recent years, because the understanding of epigenetic regulation of plant growth and development could allow the creation of new genetic variations of plants that improve crop productivity, as well as plant adaptation to stress environments [68]. Many studies on the dynamic changes in the level of methylation and function of *DME* genes in plants have been reported. In this study, we collected 39 *DME* genes from nine different plant species, including pear, and constructed a phylogenetic tree to show their relationship (Figure 2). *DME* genes are a small family in plants; there are four in *Arabidopsis*, At2g36490 (*AtROS1*), At5g04560 (*AtDME*), At3g10010 (*AtDML2*), and At4g34060 (*AtDML3*), and they are distributed into three clades; two members in *B. rapa*, one in a ROS1-DML1-like clade, and one in a DML3-like clade; two in *M. truncatula*, all classified into a ROS1-DML1-like clade; three in grape, with one in a ROS1-DML1-like clade and two in a DME-like clade; four, four, and seven in peach tomato and apple, respectively, which were distributed in three clades like those in *Arabidopsis* and pear (Figure 2).

The *DME* genes distribution across different species varies, as do the genes functions. The ROS1 plays important roles in different developmental stages and responses to stress environments in plants. Studies have shown that *AtROS1* plays a role in UV-B induced- and oxidative DNA damage [66]; *AtDME* is required for endosperm gene imprinting and seed viability [69], and *SlDML2* is critical for tomato fruit ripening [10]. Using the information that we have regarding gene functions in the model plant *Arabidopsis* and other closely related *Rosaceae* plants, we can carry out functional analysis in pear and its root stock *P. betulaefolia*. This will provide valuable information for further research on *DME* genes in *P. betulaefolia*.

### 4.3. *PbDME* Genes are Evolutionarily Conserved in Rosaceae Plants

WGD, which eventually results in massive silencing and elimination of duplicated genes, has long been recognized as a significant force in plant evolution [70]. Previous studies have indicated that a WGD event occurred about 30~45 million years ago in an ancestor of pear and apple, prior to the divergence of these two taxa [49]. In the present study, seven and seven *DME* genes were identified in the pear and apple genomes, respectively, which had both been subjected to the same WGD event that affected the ancestor fruit. Among them, there were two and three pairs of *DME* genes in the pear and apple genome, respectively, which were paralogous (Figure 4 and Table S4). The estimated divergence time for the paralogous gene pairs in pear and apple occurred much earlier than the estimate for the orthologous gene pairs (Table S4). This indicates that the WGD event occurred prior to speciation, which is consistent with the premise that the WGD occurred prior to the divergence of pear and apple. Interestingly, as many as seven *DME* family genes were found in the peach genome, which has not undergone a recent WGD [71]. Most of the *DME* genes in pear and apple that were products of the WGD event were preserved, which could be due to the crucial roles that they play in the growth and development, as well as the responses to environmental conditions, of pear and apple. Research on the relationship between homologous *DME* gene pairs in pear provides a unique perspective on the evolution of Rosaceae plants.

### 4.4. Alteration of DNA Methylation Level is Essential to Salt Response in *P. betulaefolia*

Growing evidence from recent studies has indicated that changes in DNA methylation levels play crucial roles in the regulation of stress responses and adaptation in plants [72,73]. The level of DNA methylation is dynamically regulated in plants when they are exposed to salt stress conditions [26,74]. *DME* genes also play important roles in plants’ response to environments. Studies conducted with maize showed that the specific demethylation of genes is an active and rapid epigenetic response to cold stress in maize during the seedling stage, further elucidating the mechanisms of maize’s adaptation to cold stress [75]. In this study, the expression of *PbDME* genes was altered in response to a salt stress treatment, and concomitantly, some of the examined salt-responsive genes were differentially methylated in their promoter regions (Figure 6 and Table 2). This resulted in changes in the expression level of these downstream genes in *P. betulaefolia* in response to salt stress. These results indicated that the *DME* genes play an important role in the response of *P. betulaefolia* to salt stress. There was not a perfect correlation, however, between the expression level of *PbDME* genes and the methylation levels of the selected salt-responsive genes; the expressions of *PbCIPK22* and *PbCBL1* were up-regulated at 24 h after salt stress treatment, which was consistent with the declining level of demethylated promoter regions (Figure S6). In another case, the *PbCIPK3* gene was induced by salt stress, but without any direct relationship with the methylation level.

On the other hand, some of the *DME* genes were repressed under salt stress (Figure 6), which indicated that there are other genes involved in salt response in *P. betulaefolia*. In some cases, a high methylation level increased plants’ tolerance. For example, higher global methylation levels have been observed in salt tolerant cottons than salt sensitive cottons [29]. Salt marsh (SM) plants were hypomethylated (14.6% of loci had methylated samples) in comparison to riverside (RS) (32.1% of loci had methylated samples) [76]. These studies show that complex gene expression alterations occur while responding to salt stress, or that multiple signaling pathways exist in *P. betulaefolia* that are involved in salt stress response. Although the functional mechanisms of the *PbDME* genes underlying their role in the response to salt stress remain unclear, the results of our study have provided a new perspective on the epigenetics of salt stress responses in pear, and perhaps other temperate tree fruit crops.

### 4.5. Excellent Candidates for Pear Improvement

A growing percentage of agricultural land worldwide is affected by high salinity, due to both natural causes and current agricultural practices [77]. Salinity poses two major threats to plant growth: ions outside the root cause osmotic stress, which is similar to the stress caused by drought, while ions that enter the plant cause ionic stress (e.g., Na^+^ or Cl^−^ toxicity). How plants deal with salinity and how successful their strategies are various widely among species [77,78]. In the present study, we identified some important candidate genes that were highly or specifically expressed in some tissues, such as genes *PbDME3–7*, which were dominantly expressed in roots (Figure 5). All of them were responsive to salt stress at different time points or, in some cases, repressed (Figure 6). These genes are potential candidates for studying the functions of *PbDME* genes in the roots, in response to different environments and in epigenetics research on *P. betulaefolia*. Furthermore, these genes provide excellent clues to improve the salt response in pear breeding.

## 5. Conclusions

In the present study, putative *DME*s in the pear genome were identified and analyzed. Seven *DME* family genes were found in the pear genome and could be classified as *ROS1*- and *DML2*-like, *DME*-like, and *DML3*-like groups, based on the organization of various characteristic domains. The identification and classifications were supported by structural characteristics of the genes and proteins, as well as by phylogenetic analysis. The structure of the pear *DME* genes was relative complex and the DME7 proteins did not contain the Perm_CXXC domain. Based on *K*s values, the MTase family genes in pear was subjected to a WGD that occurred in the ancestral ancestor of pear and apple genome prior to their divergence. Expression profiles indicated that high salinity stress induced the expression of DMEs and changed the methylation levels of salt-responsive genes in *P. betulaefolia*. This study provided a genome-wide survey of the *DME* gene family in pear and highlighted their roles in salt response in *P. betulaefolia* under salt stress. These results suggest that *PbDME* genes play a role in the response to salt stress. The present study provides a foundation for understanding the complex functions of the *DME* gene family in pear, and it will facilitate epigenetic studies for the response of pear to salt stress.

## Figures and Tables

**Table 1:** The characteristics of demethylase (DME) family members in Pear.

Gene Name	Gene ID	Position	No. of Intron	CDS (bp)	Size (aa)	MW (kDa)	pI	*Arabidopsis* Ortholog
*PbDME1*	Pbr016811.1	Chr14:3306106–3315557(−)	18	5433	1810	202.43	5.89	AT2G36490/AT3G10010
*PbDME2*	Pbr005494.1	Chr12:20839378–20849041(+)	18	5406	1801	201.51	5.95	AT2G36490/AT3G10010
*PbDME3*	Pbr014401.1	Chr9:6995060–7006208(−)	20	6048	2015	225.75	6.38	AT5G04560
*PbDME4*	Pbr025523.1	scaffold404.0:407609–416687(−)	19	5778	1925	216.71	8.52	AT5G04560
*PbDME5*	Pbr025515.1	scaffold404.0:259775–268853(−)	19	5778	1925	216.71	8.52	AT5G04560
*PbDME6*	Pbr038018.2	Chr16:14869003–14879562(−)	19	5880	1959	220.49	7.20	AT5G04560
*PbDME7*	Pbr027983.1	Chr11:19457199–19465105(+)	19	3702	1233	138.88	9.01	AT4G34060

Note, CDS: coding sequence; MW: molecular weight; pI: isoelectric point; pI: isoelectric point.

**Table 2:** Methylation analysis of a 200 bp promoter segment in *PbCIPK1*, *PbCIPK20*, and *PbCIPK22* in *P. betulaefolia* under salt stress treatment.

***PbCIPK1***	**Length of Target Genome Sequence (204 bp)**				**Number of Bisulfite Sequences (Used/Excluded/Total)**																	
	**Number of CpGs (7)**				**7/0/7**																	
	**Number of Sites**	1	2	3	4	5	6	7	8	9	10	11	12	13	14	15	16	17	18	19	20	
	**CpG Positio**	11	37	48	63	74	80	85	– –	– –	– –	– –	– –	– –	– –	– –	– –	– –	– –	– –	– –	Total
	**Controls Me-CpG**	0/10	0/10	0/10	1/10	0/10	0/10	0/10	– –	– –	– –	– –	– –	– –	– –	– –	– –	– –	– –	– –	– –	**1/70**
		0.0%	0.0%	0.0%	10.0%	0.0%	0.0%	0.0%	– –	– –	– –	– –	– –	– –	– –	– –	– –	– –	– –	– –	– –	**1.4%**
	**NaCl Me-CpG**	0/10	0/10	0/10	0/10	0/10	0/10	0/10	– –	– –	– –	– –	– –	– –	– –	– –	– –	– –	– –	– –	– –	0/70
		0.0%	0.0%	0.0%	0.0%	0.0%	0.0%	0.0%	– –	– –	– –	– –	– –	– –	– –	– –	– –	– –	– –	– –	– –	0.0%
***PbCIPK20***	**Length of Target Genome Sequence (225 bp)**																					
	**Number of CpGs (20)**				**20/0/20**																	
	**CpG Position**	4	10	15	44	53	67	76	86	97	100	110	122	129	141	144	164	179	207	222	224	Total
	**Controls Me-CpG**	0/10	0/10	0/10	0/10	0/10	0/10	1/10	0/10	0/10	0/10	0/10	0/10	0/10	0/10	0/10	0/10	0/10	0/10	0/10	0/10	**1/200**
		0.0%	0.0%	0.0%	0.0%	0.0%	0.0%	10.0%	0.0%	0.0%	0.0%	0.0%	0.0%	0.0%	0.0%	0.0%	0.0%	0.0%	0.0%	0.0%	0.0%	**0.5%**
	**NaCl Me-CpG**	0/10	0/10	0/10	0/10	0/10	0/10	0/10	0/10	0/10	0/10	0/10	0/10	0/10	0/10	0/10	0/10	0/10	0/10	0/10	0/10	0/200
		0.0%	0.0%	0.0%	0.0%	0.0%	0.0%	0.0%	0.0%	0.0%	0.0%	0.0%	0.0%	0.0%	0.0%	0.0%	0.0%	0.0%	0.0%	0.0%	0.0%	0.0%
***PbCIPK22***	**Length of Target Genome Sequence (215 bp)**																					
	**Number of CpGs (11)**				**11/0/11**																	
	**CpG Position**	1	5	27	29	108	140	159	170	179	198	214	– –	– –	– –	– –	– –	– –	– –	– –	– –	Total
	**Controls Me-CpG**	0/10	0/10	0/10	0/10	0/10	1/10	0/10	0/10	0/10	0/10	0/10	– –	– –	– –	– –	– –	– –	– –	– –	– –	**1/110**
		0.0%	0.0%	0.0%	0.0%	0.0%	10.0%	0.0%	0.0%	0.0%	0.0%	0.0%	– –	– –	– –	– –	– –	– –	– –	– –	– –	**0.9%**
	**NaCl Me-CpG**	0/10	0/10	0/10	0/10	0/10	0/10	0/10	0/10	0/10	0/10	0/10	– –	– –	– –	– –	– –	– –	– –	– –	– –	0/110
		0.0%	0.0%	0.0%	0.0%	0.0%	0.0%	0.0%	0.0%	0.0%	0.0%	0.0%	– –	– –	– –	– –	– –	– –	– –	– –	– –	0.0%

Note: 10 replicates per DNA sample were analyzed. The percentage of CpG methylation level is highlighted.

## Supplementary Materials

**Figure S1**: Diagram of the RNA integrity/quality and qPCR primer efficiency. **Figure S2**: Alignments of PbDME family proteins. **Figure S3**: Conserved motif analysis of PbDME family proteins. **Figure S4**: Expression patterns of DME genes in different organs in *Arabidopsis*. **Figure S5**: Expression patterns of DME genes under salt stress in *Arabidopsis* roots. **Figure S6**: Expression profiles of salt-responsive genes in *Pyrus betulaefolia*. **Table S1**: Primers used in this paper. **Table S2**: GO analysis for the *PbDME* genes. **Table S3**: Conserved motif analysis of PbDME family proteins. **Table S4**: Orthologous and paralogous gene pairs in the pear and apple DME family.
