# Supplementary material for: Genome-Wide Characterization of DNA Demethylase Genes and Their Association with Salt Response in Pyrus
Source: Genes (Basel). 2018 Aug 6;9(8):398. doi: 10.3390/genes9080398 (PMC6116010; doi:10.3390/genes9080398)
Supplement: Supplementary file 1 [file genes-09-00398-s001.zip › Captions for supplementary files.docx]

**Captions for Supplementary Materials:**

**Figure S1**: Diagram of the RNA integrity/quality and RT-qPCR primer efficiency. (a), PAGE results showed the specificity of the primers, with the primers used in this study marked in stars. (b), RNA integrity/quality.

**Figure S2**: Alignments of PbDME family proteins. Sequences in the boxes are the DNA demethylase domains, a predicted RRM-DME domain in the red box, and a single unit of the Perm_CXXC domain in the blue box.

**Figure S3**: Conserved motif analysis of PbDME family proteins.

**Figure S4**: Expression patterns of DME genes in different organs in *Arabidopsis*.

**Figure S5**: Expression patterns of DME genes under salt stress in *Arabidopsis* roots.

**Figure S6**: Expression profiles of salt-responsive genes in *Pyrus betulaefolia*. Roots of young pear plants (approximately eight leaves-old) were exposed to 200 mM NaCl by watering the plants with the salt solution. Roots (R) (a), stems (S) (b) and leaves (L) (c) were sampled at 0, 12, 24, 48, and 72 hr and relative expression was determined by RT-qPCR analysis. Relative expression was calculated using the 2−∆ ∆ CT method.

**Table S1**: Primers used in this paper.

**Table S2**: GO analysis for the *PbDME* genes.

**Table S3**: Conserved motif analysis of PbDME family proteins.

**Table S4**: Orthologous and paralogous gene pairs in the pear and apple DME family.
