# Supplementary figures and images for: Genome-Wide Characterization of DNA Demethylase Genes and Their Association with Salt Response in Pyrus
Source: Genes (Basel). 2018 Aug 6;9(8):398. doi: 10.3390/genes9080398 (PMC6116010; doi:10.3390/genes9080398)

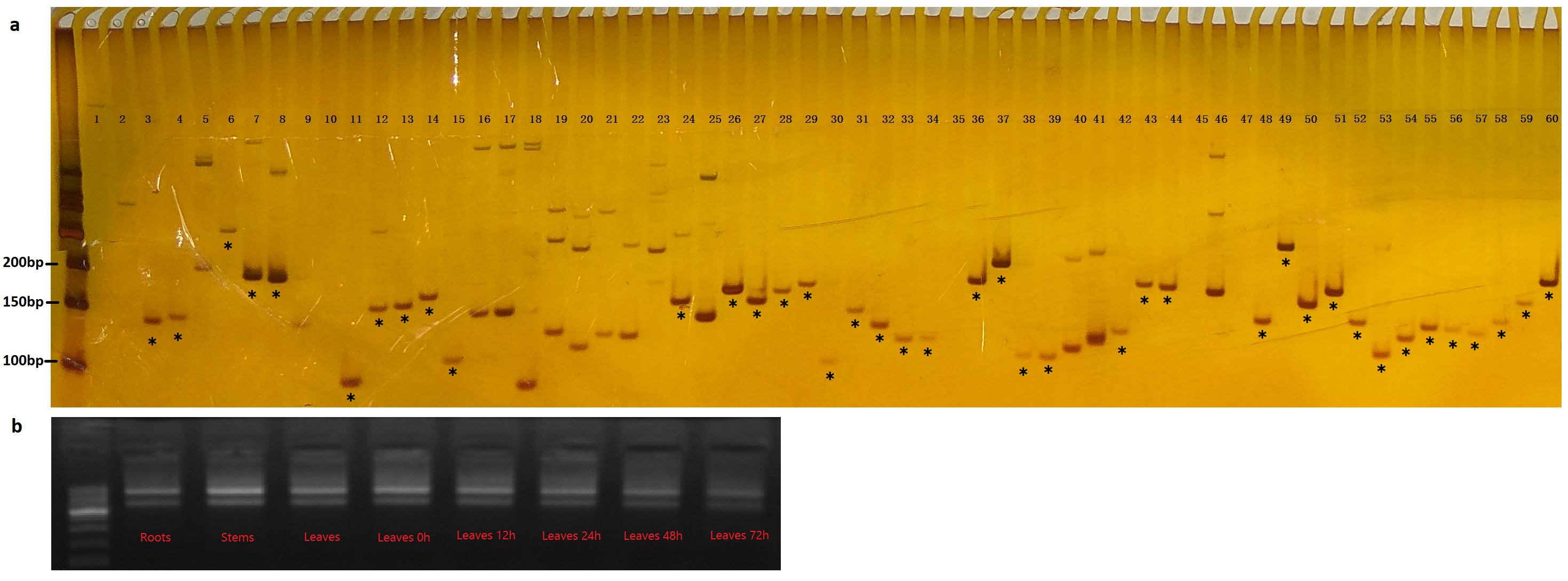

Supplement: Supplementary file 1 [file genes-09-00398-s001.zip › Fig. S1.tif]

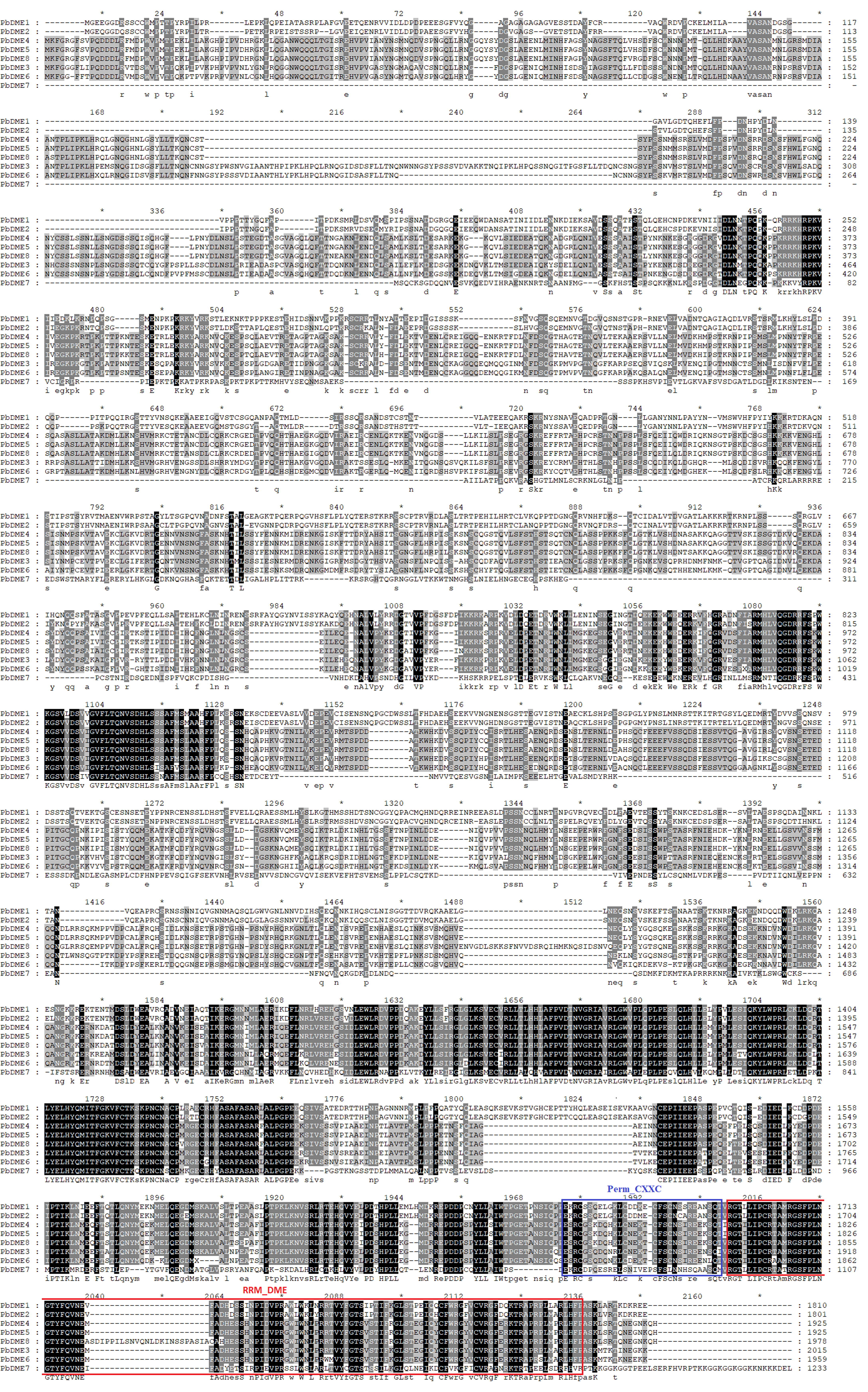

Supplement: Supplementary file 1 [file genes-09-00398-s001.zip › Fig. S2.tif]

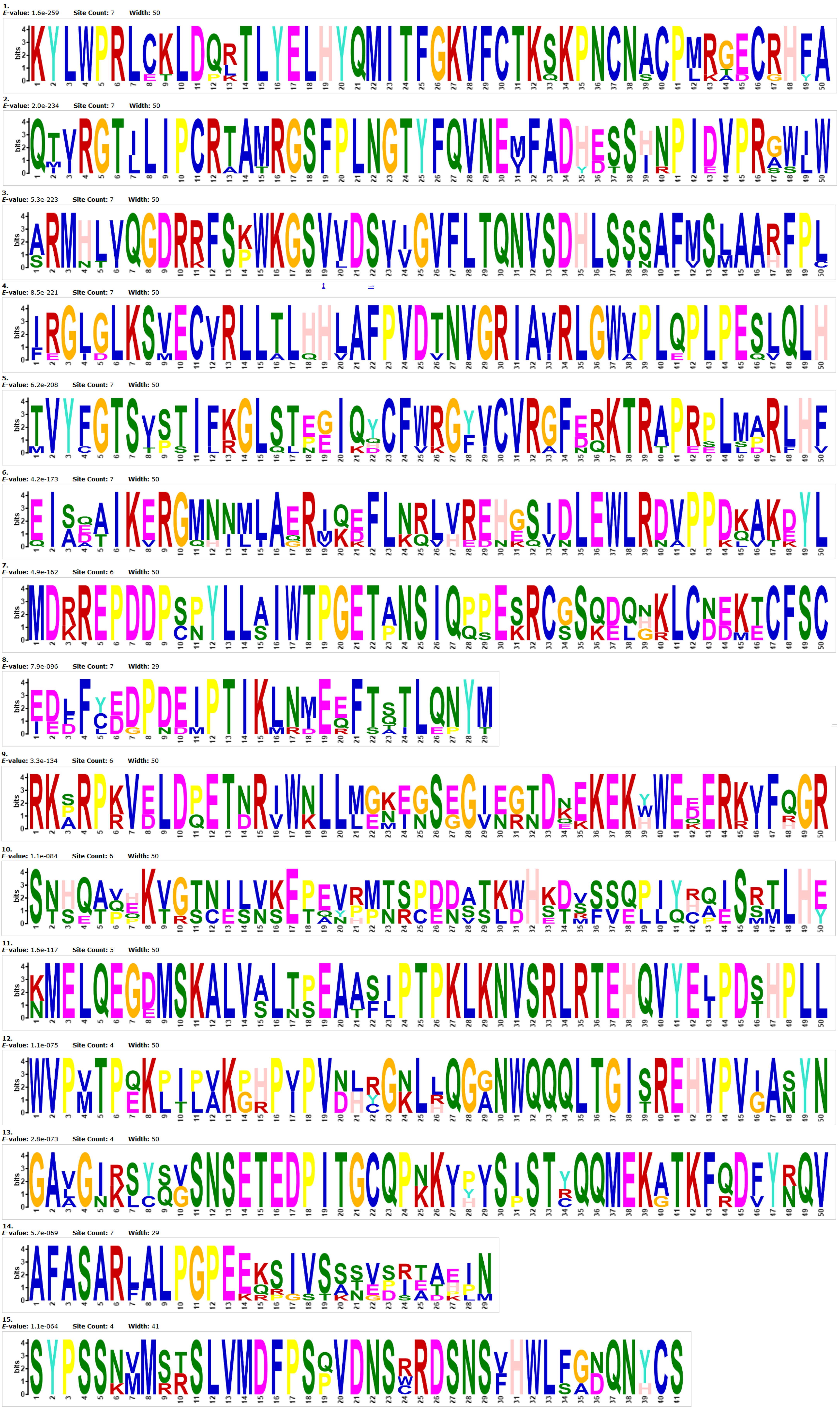

Supplement: Supplementary file 1 [file genes-09-00398-s001.zip › Fig. S3.tif]

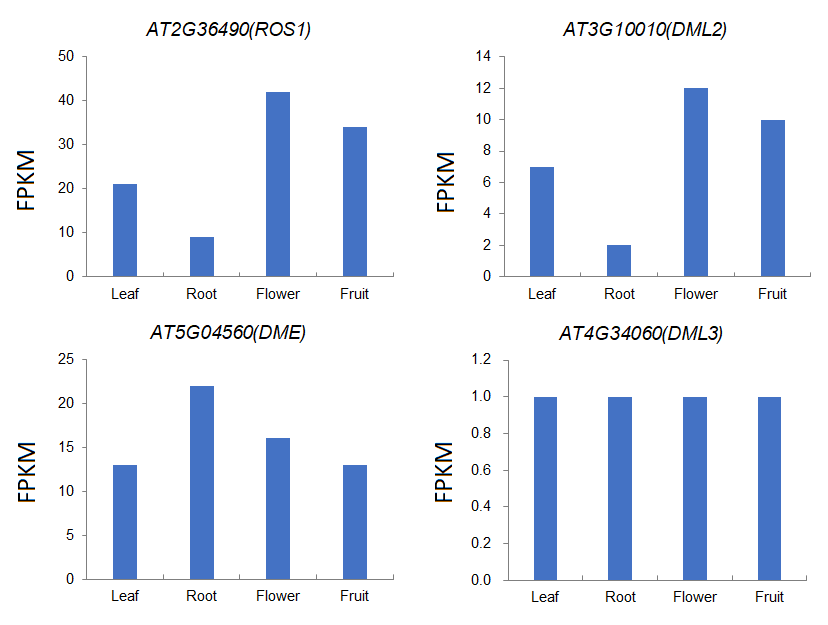

Supplement: Supplementary file 1 [file genes-09-00398-s001.zip › Fig. S4.tif]

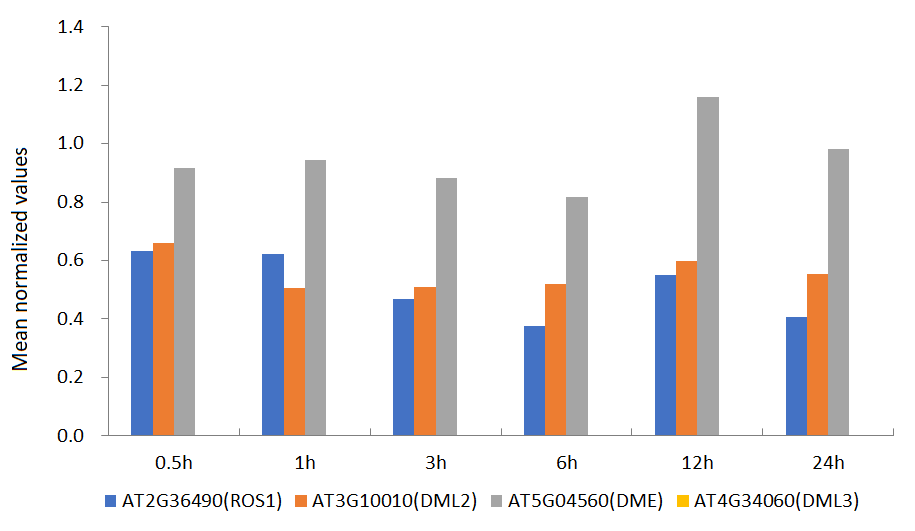

Supplement: Supplementary file 1 [file genes-09-00398-s001.zip › Fig. S5.tif]

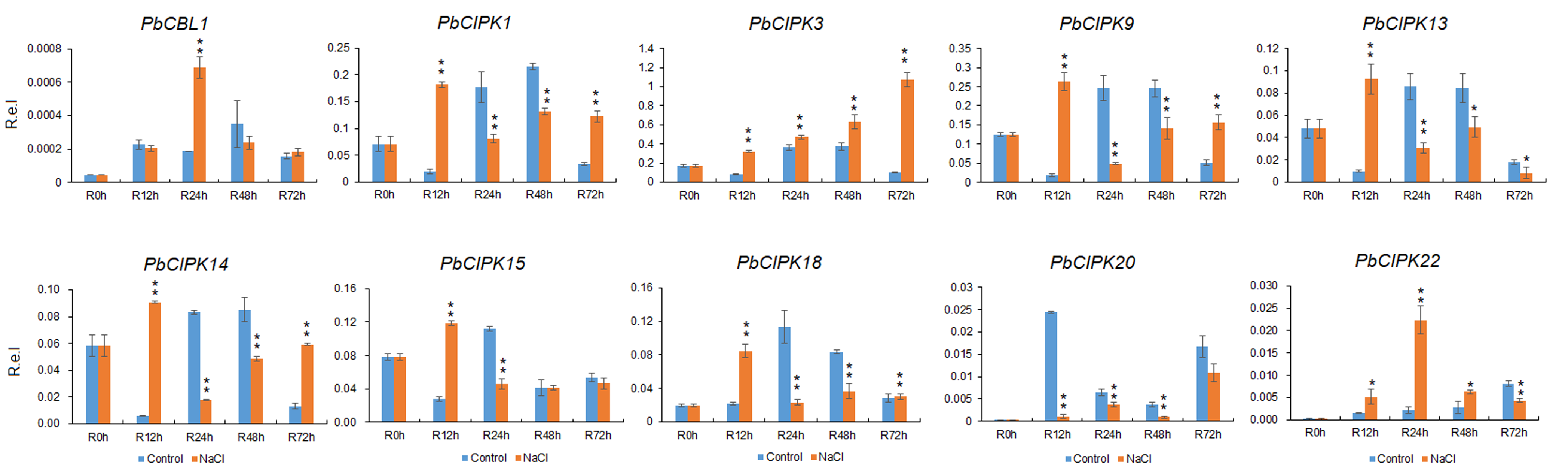

Supplement: Supplementary file 1 [file genes-09-00398-s001.zip › Fig. S6.tif]
